# Optimizing Dietary Habits in Adolescents with Polycystic Ovary Syndrome: Personalized Mediterranean Diet Intervention via Clinical Decision Support System—A Randomized Controlled Trial

**DOI:** 10.3390/children11060635

**Published:** 2024-05-24

**Authors:** Alexandra Foscolou, Panos Papandreou, Aristea Gioxari, Maria Skouroliakou

**Affiliations:** 1 Department of Nutritional Science and Dietetics, School of Health Sciences, 24100 Kalamata, Greece; alexandra.foscolou@go.uop.gr; 2 Department of Nutrition, IASO Hospital, 37-39 Kifissias Ave., 15123 Athens, Greece; 3 Department of Dietetics and Nutritional Science, School of Health Science and Education, Harokopio University, 70 El. Venizelou Ave., 17676 Athens, Greece; mskour@hua.gr

**Keywords:** polycystic ovary syndrome, clinical decision support system, adolescents, females, Mediterranean diet, anxiety

## Abstract

The hypothesis of this randomized controlled trial was that a clinical decision support system (CDSS) would increase adherence to the Mediterranean diet (MD) among adolescent females with polycystic ovary syndrome (PCOS). The objective was to assess the impact of personalized MD plans delivered via a CDSS on nutritional status and psychological well-being. Forty adolescent females (15–17 years) with PCOS were randomly assigned to the MD group (n = 20) or the Control group (n = 20). The MD group received personalized MD plans every 15 days via a CDSS, while the Control group received general nutritional advice. Assessments were conducted at baseline and after 3 months. Results showed significantly increased MD adherence in the MD group compared to the Control group (*p* < 0.001). The MD group exhibited lower intakes of energy, total fat, saturated fat, and cholesterol, and higher intakes of monounsaturated fat and fiber (*p* < 0.05). Serum calcium and vitamin D status (*p* < 0.05), as well as anxiety (*p* < 0.05) were improved. In conclusion, tailored dietary interventions based on MD principles, delivered via a CDSS, effectively manage PCOS in adolescent females. These findings highlight the potential benefits of using technology to promote dietary adherence and improve health outcomes in this population. ClinicalTrials.gov registry: NCT06380010.

## 1. Introduction

Based on statistics from the World Health Organization (WHO), approximately 1 out of 10 (8–13%) of reproductive-aged females suffer from polycystic ovary syndrome (PCOS), while, globally, 7 out of 10 affected females remain undiagnosed [1]. PCOS is a multifaceted and diverse endocrine disorder with uncertain etiology. However, compelling evidence suggests that it mostly arises from the interplay of genetic, metabolic, and environmental influences [2]. Females with PCOS exhibit distinct characteristics such as acne, irregular menstrual periods, and excessive growth of body hair caused by increased levels of testosterone in the bloodstream. Hypertriglyceridemia has been found to be a determinant of hyperandrogenism in PCOS patients [3]. Additionally, symptomatology includes increased body weight and significant manifestations of depression or anxiety [4]. Deficiency of vitamin D has also been identified in PCOS patients, which may partly explain the hormonal and metabolic dysregulation seen in PCOS [5]. Vitamin D plays an important role in calcium homeostasis, which is crucial for the growth and development of young patients with PCOS [6]. It is noteworthy that disruptions in calcium balance may lead to the growth of ovarian follicles facilitating the progression of PCOS [7]. In fact, about the same proportion of adolescent girls as that of all reproductive-aged females encounter this disorder around the world [8]. The period of adolescence is considered to be a crucial stage of development, during which individuals acquire lifestyle behaviors that hold significance for their overall health [9]. The presence of PCOS in teenagers poses distinct difficulties as it affects their physical, reproductive, and psychological well-being.

Multiple studies have documented a link between PCOS and lifestyle [10,11,12]. Due to this rationale, a multidimensional approach has been proposed to enhance the management of PCOS, encompassing interventions targeting external factors that may influence PCOS, such as adoption of healthy eating habits and lifestyle ones [13]. However, based on the 2023 International PCOS Guidelines, there is no evidence to substantiate the superiority of any certain dietary composition in terms of anthropometric, metabolic, hormonal, reproductive, or psychological outcomes [14]. Additionally, since health advantages may come from any diet that meets population norms, it is highly recommended that healthcare practitioners recommend sustainable healthy eating that matches individual preferences [14].

A sustainable healthy dietary pattern that could be considered is the Mediterranean diet (MD). The MD is well recognized as a highly regarded nutritional pattern for its multiple health advantages [15,16] due to the abundance of beneficial nutrients present in the included foods. Multiple studies emphasize the preventive function of MD in relation to obesity, cardiometabolic abnormalities, and the development of chronic non-communicable diseases, and other neurodegenerative disorders [17]. In short, the MD embodies an all-encompassing dietary approach that not only provides nourishment to the body but also promotes general well-being.

The MD may also have beneficial effects on PCOS. In a recent case–control study including 112 adult women diagnosed with PCOS and 112 healthy controls, PCOS women consumed less complex carbohydrates, dietary fiber, monounsaturated fatty acids (MUFAs), and polyunsaturated fatty acids (PUFAs), as well as higher saturated fatty acids (SFA) than the controls [18]. In fact, women with PCOS showed higher fat mass and less fat-free mass than their healthy counterparts, while adherence to the MD was negatively correlated with testosterone levels in the whole sample [18]. Nevertheless, randomized controlled trials (RCTs) investigating the effects of MD on PCOS management are lacking. Recently, Mei et al. showed a significant reduction in body weight (BW) and body fat mass of overweight PCOS women following a combination of the MD and a low-carbohydrate diet for 3 months, compared to a low-fat diet [19].

There is evidence that the increase in MD adherence can be supported by the use of clinical decision support systems (CDSS) [20,21,22]. CDSSs have the potential to be extremely useful and beneficial tools in clinical practice when they are designed and executed correctly [20]. One of the main aims of CDSSs is to encourage compliance with lifestyle guidelines aimed at healthy weight management [23]. Dietary interventions using software-based tools for 3 months revealed beneficial effects on adolescent girls with PCOS, improving inflammatory status and anthropometrical parameters [24].

Thus, the current trial aimed to investigate, over a 3-month intervention period, the potential benefits of integrating a CDSS into dietary practices for improving nutritional status and alleviating health-related anxiety in adolescent girls diagnosed with PCOS. Hence, the objective was, primarily, to examine whether the adherence to the MD in adolescent girls with PCOS would be enhanced with the assistance of a CDSS. Secondarily, we would like to explore the effects of MD on the nutritional and anxiety status of females with PCOS as indicated by anthropometry, dietary intake of nutrients, and hematology assessment including triglycerides (TG), vitamin D, and calcium status.

## 2. Materials and Methods

### 2.1. Ethics

The study underwent assessment and received approval from the Ethics Committee of IASO HOSPITAL in Athens, Greece (Approval Code #1g310519). The trial adhered to the guidelines set forth in the Helsinki Declaration (1964) and followed the standards of Good Clinical Practice. ClinicalTrials.gov (accessed on 10 April 2024) registry: NCT06380010.

### 2.2. Participants

All volunteers were outpatients of the IASO HOSPITAL in Athens, Greece. The initial recruitment of adolescents with PCOS was conducted through written announcements at the clinic facilities and the hospital’s official website. Female participants, who expressed their willingness to take part, attended personal meetings with the appointed dietitians who provided comprehensive explanations on the objectives, methods, and the possible benefits and risks of the trial. All patients were given a pamphlet containing relevant information. Prior to the commencement of the recruitment process, every eligible volunteer, and their guardian, executed an informed written agreement and subsequently retained a physical copy of the executed document. All eligible patients were from Athens (Greece) and were recruited in December 2019.

**Inclusion criteria**

(a) Newly diagnosed with PCOS females < 18 years of age.
(b) Females with guardians who provided signed participation consent.

**Exclusion criteria**

(a) Females ≥ 18 years of age.
(b) Females suffering from severe illnesses (i.e., organ failure, autoimmune diseases, congenital metabolic disorders), psychiatric disease, or with emerging health issues that could hinder the trial.
(c) Pregnant or lactating females.
(d) Females diagnosed with alcoholism or drug addiction.
(e) Females being under any drug treatment.
(f) Females followed a specific type of diet within the past 5 years or have used nutrient or non-nutrient supplements in the previous half-year.
(g) Females with guardians non able to read and understand the consent information.

### 2.3. Study Design

Females who met the criteria were allocated in groups to one of either the Control group or the Intervention (MD) group using a random assignment method applied by an independent statistician. During the trial, every participant in both groups had two individual sessions with the assigned researchers. These sessions took place at the beginning of the trial and three months later, and involved anthropometry measurements, evaluation of dietary habits, assessment of psychological well-being, and collection of blood samples. Treatment allocation was not revealed to the appointed statistician until the end of the trial and release of the final outcomes.

#### 2.3.1. MD Group

Prior to the commencement of the trial, each participant was assigned to a highly skilled dietitian. The dietitian implemented an individualized daily dietary regimen in accordance with the participant’s requirements, routines, and inclinations, which was derived from the MD produced by the CDSS software (https://innovenn.com/software-as-a-medical-device-samd/clinical-decision-support-software/). As described previously [21], the CDSS software was utilized to calculate all necessary components for the synthesis of the dietary plan, including current body weight and macronutrient distribution. The dietary protocol followed by the CDSS comprised a 15-day rotation of daily meals and nutritional suggestions consistent with the “National Dietary Guidelines for Children and Adolescents” [25]. Emphasis was given to fruits, vegetables, complex carbohydrates, legumes, extra virgin olive oil, nuts, fresh fish, low-fat dairy, as well as vitamin-D rich foods (e.g., fatty fish, dairy, yogurt, cheese, juices, and cereals). The female participants obtained unique login credentials for the CDSS and received instruction on its operation from the designated dietitians. They were directed to access their CDSS account remotely on a regular basis in order to monitor their nutritional status, including weight gain and healthy eating. Participants were also directed to complete a three-day dietary journal in the CDSS on a weekly basis; this information was automatically accessible to the dietitians. Telephone interviews were conducted every other week in order to supplement the nutritional and lifestyle consultations. In addition, unanticipated phone calls requesting 24 h dietary recalls were received.

#### 2.3.2. Control Group

The Control group participants were not provided with the CDSS. They simply recieved basic lifestyle instructions based on the “National Dietary Guidelines for Children and Adolescents” [25] which are based on the principles of the MD, during biweekly phone consultations with the dietitians. The women in the Control group were directed to maintain a food diary for three consecutive days each week, which they then transmitted to the designated dietitian via email. Once again, unsolicited phone calls were conducted in order to acquire 24 h dietary recalls.

### 2.4. Measurements

#### 2.4.1. Anthropometric Characteristics

Τhe air displacement plethysmography method for research and clinical applications (BOD POD^®^ Body Composition Tracking Systems, Life Measurement, Inc., Rome, Italy) [26] was used to measure the participants’ current body weight and body fat mass percentage at the beginning and at the trial endpoint (3 months). The measurement of height was conducted using a calibrated stadiometer with an accuracy of 0.1 cm. The weight status of participants was assessed using the age- and sex-specific body mass index cut-off criteria established by the International Obesity Task Force (IOTF) [27]. Subsequently, they were classified into three categories: underweight, normal-weight, and overweight/obese.

#### 2.4.2. Dietary Habits Assessment and Lifestyle Characteristics

The estimation of nutrient intake was conducted using the Diet Analysis Plus program (version 6.1, Esha Research, Salem, MA, USA) based on the collected food diaries and 24 h dietary records. The adherence to the Mediterranean diet was assessed using the KIDMED, which is a quality indicator specifically designed for children and adolescents [28]. Dietary habits that exhibit a favorable aspect to this dietary pattern were assigned a score of +1. Dietary habits that have an unfavorable association were assigned a score of −1. Dietary habits that have a neutral association were assigned a score of 0. The theoretical maximum score spans from −4 to 12. Lower scores corresponded to a lack of commitment to the Mediterranean diet, whereas higher values showed a strong devotion to the Mediterranean diet.

#### 2.4.3. Anxiety Assessment

The Hospital Anxiety and Depression Scale (HADS) was used in this trial, consisting of 14 self-assessed questions [29,30]. Among these questions, seven were specifically designed to measure depression, while the remaining seven measured anxiety. The scoring system for each category ranges from 0 to 21, with values over “7” indicating potential instances of depression or anxiety, respectively [29].

#### 2.4.4. Blood Sample Collection

Blood samples (about 15–20 mL) were collected from each patient at 0 and 3 months after an overnight fasting period, using a catheter inserted into an antecubital vein. Plasma separation was performed using blood collection tubes that contained ethylenediamine tetraacetic acid (EDTA). The sera were isolated by allowing whole blood samples to coagulate at ambient temperature. The centrifugation was set to 3000 rpm for 10 min at a temperature of 4 °C. All tests were conducted using freshly isolated serum samples.

The serum triacylglycerol levels were determined using a biochemical analyzer (Cobas 8000 modular analyzer, Roche Diagnostics GmbH, Mannheim, Germany). The measurement of serum 1,25-dihydroxyvitamin D [1,25(OH)2D] (vitamin D) was conducted using an automated chemiluminescence system (Cobas e 801 analytical module, Roche Diagnostics GmbH, Mannheim, Germany). Serum calcium was quantified using the integrated clinical chemistry and immunoassay analyzer, Dimension^®^ EXL™ 200 Integrated Chemistry System (Siemens Healthcare GmbH, Erlangen, Germany) using manufacturer’s reagents. Optimal calcium levels are 9–11 mg/dL.

### 2.5. Primary Outcome and Sample Size Calculation

Changes in the adherence to the Mediterranean diet, as measured by the KIDMED score, constituted the principal outcome of the research. Modifications in anthropometric measurements, blood parameters, nutritional consumption, and the Hospital Anxiety and Depression Scale were among the secondary outcomes. A sample size of at least 32 patients, with 16 patients in each group, was deemed adequate to yield a statistically significant difference of 1 in KIDMED (standard deviation of mean = 1). This conclusion was reached using a two-tailed *t*-test with 80% power and a 5% level of significance.

### 2.6. Statistical Analysis

The continuous variables were reported as mean values with the standard deviation of the mean (SD) for normally distributed variables, and the median plus interquartile range (IQR) for variables that were not normally distributed. The categorical variables were displayed as absolute and relative frequencies (%). The normality of the distribution of continuous data was assessed by both graphical and statistical methods (Shapiro–Wilk test). Student’s *t*-test was employed to compare the mean changes between the Control and MD groups for variables that were normally distributed. For variables that were not normally distributed, the Mann–Whitney U test was utilized. Statistical significance was set at *p*-value < 0.05. The SPSS statistical package (version 29.0, SPSS, Inc., IBM, Chicago, IL, USA) was used.

## 3. Results

**Figure 1:**
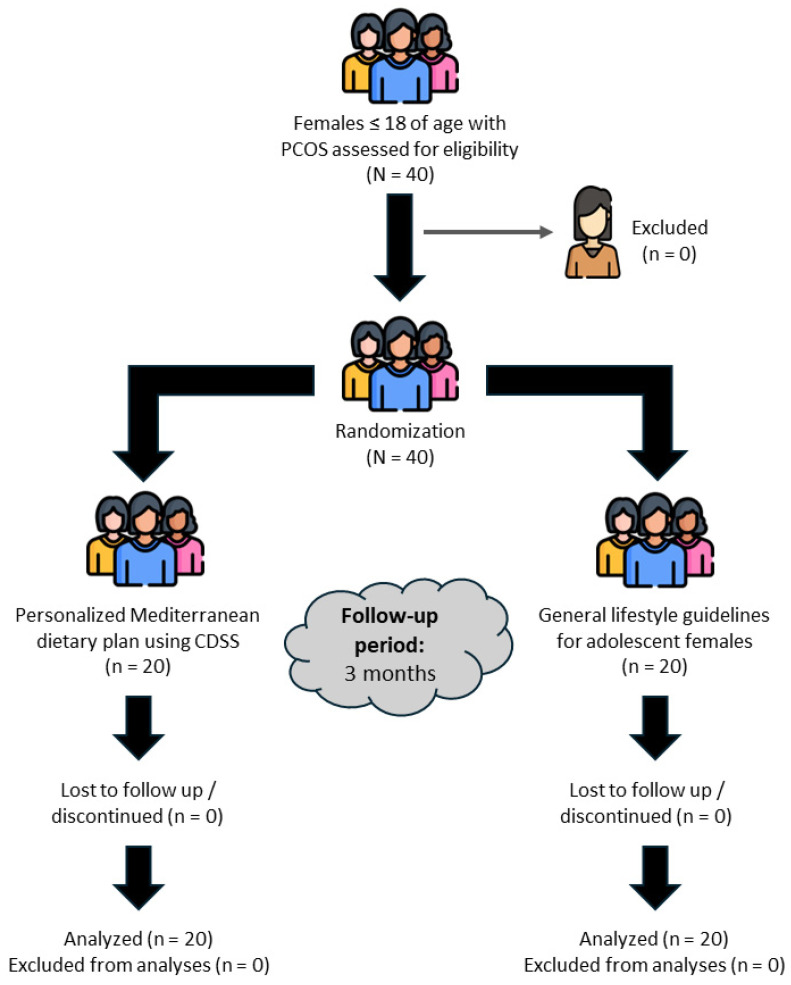
CONSORT flow diagram. N: total sample size; n: number of subjects in each subgroup, CDSS: Clinical Decision Support System.

As shown in Figure 1, a total of 40 eligible females participated in the trial. Participants were separated into two groups of equal size and characteristics: the Control (n = 20) and the MD (n = 20) group.

Table 1 presents the participants’ baseline characteristics. As depicted, no statistically significant differences were noted between the two groups (all *p*’s > 0.05). The mean age of the participants was 16.4 and they all had normal weight. Moreover, a non-negligible percentage of participants from both groups had anxiety symptoms, as derived from HADS score.

**Figure 2:**
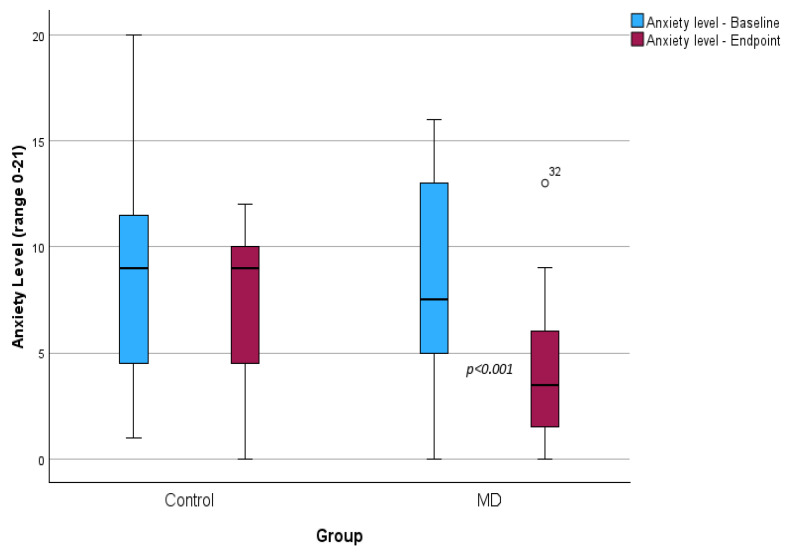
Boxplot depicting psychological well-being using HADS at baseline and 3 months after the intervention.

In Table 2 and Table 3 and Figure 2, the impact of CDSS intervention on anthropometry, blood indicators, diet consumption, and psychological well-being at both baseline and follow-up, as well as the comparison between the two groups, are depicted.

Alterations seen in anthropometric parameters and blood indicators are displayed in Table 2. In brief, body weight decreased significantly in both groups after 3 months, whereas fat mass (*p* < 0.001) and triacylglycerols (*p* = 0.002) decreased only in the MD group. Moreover, serum vitamin D increased significantly in both groups (all *p*’s < 0.001) with a comparatively greater increase in the MD group (*p* = 0.011). Accordingly, after the three-month intervention in the adolescent girls, an increase in serum calcium levels was revealed in the MD group (*p* = 0.002) but not in the Control group (*p* > 0.05).

Modifications in food consumption after the 3-month intervention are shown in Table 3. As depicted, adolescent girls in the MD group increased the level of adherence to the MD after the intervention (*p* < 0.001), contrary to the girls in the Control group (*p* = 0.48). The only group that encountered a decrease in caloric intake, fat, saturated fatty acids and dietary cholesterol, and an increase in monounsaturated fatty acids was the MD group (all *p*’s < 0.05). Conversely, the Control group experienced a rise in both fat and dietary cholesterol intake (all *p*’s < 0.001). Meanwhile, fiber intake was increased in both groups (*p*’s < 0.05); however, the rise in the MD group seemed to be more pronounced compared to the Control group.

Finally, the assessment of psychological well-being using the HADS scale is displayed in Figure 2. The MD group experienced a statistically significant reduction in anxiety levels (8.5 ± 4.9 vs. 4.1 ± 3.4, *p* < 0.001), whereas no statistically significant reduction was observed in the Control group (8.8 ± 4.9 vs. 7.2 ± 3.6, *p* = 0.079). As depicted, at the trial endpoint, anxiety levels significantly decreased in the MD group compared to the Control group (4.1 ± 3.4 vs. 7.2 ± 3.6, *p* = 0.004).

## 4. Discussion

In this randomized controlled clinical trial, we focused on the efficacy of a personalized Mediterranean diet intervention administered through a CDSS in improving the level of adherence to the MD and anxiety status in adolescents with PCOS. The findings revealed that the MD group demonstrated favorable alterations in several health indicators associated with PCOS management and overall well-being, including enhancements in the adherence to the MD, body composition, anxiety levels, and lipid profile. The tailored nutritional counsel via the CDSS is shown to be superior to the standard treatment received by the Control group. Although both groups showed some improvements during the 3-month period, the MD group exhibited more significant and extensive changes in various areas, highlighting the significance of customized dietary treatments in managing PCOS.

It has already been said that women with PCOS have a direct correlation between their level of adherence to the MD and the severity of their condition [18]. This correlation suggests that the foods and nutrients found in the MD may have favorable impacts on the PCOS, potentially by reducing inflammation, among others. Additionally, the association between the level of adherence to the MD and fat-free mass in women with PCOS is already established [18]. Recently, it has been demonstrated that overweight PCOS patients can benefit by following a MD along with a low-carbohydrate dietary model, by restoring the menstrual cycle, enhancing physical measurements, and rectifying the disrupted endocrine system [19]. Thus, the importance of adopting the MD for adult females with PCOS has already been clarified. Hence, we managed to add to the current knowledge by combining the principles of the Mediterranean diet with a CDSS, the provided benefits could be further enhanced. In short, in the present intervention, we managed to successfully confirm the hypothesis that the implementation of a CDSS leads to a higher level of adherence to the MD and the maintenance of a healthy body weight. Adolescent girls diagnosed with PCOS in the MD group experienced a notable increase in KIDMED score following a 3-month intervention. In contrast, adolescent girls in the Control group who were given basic lifestyle instructions did not observe any such gain. As this is the first trial to use a CDSS to improve the eating habits of adolescent females diagnosed with PCOS, comparisons with other studies are not possible; however, CDSSs have already demonstrated their significance in the classification of PCOS [31]. Meanwhile, our team conducted several randomized controlled trials (RCTs) that showed the comparable influence of CDSS implementation on the adherence to the MD. These RCTs encompassed patients with breast cancer [20], multiple sclerosis [32], as well as healthy pregnant women [21].

Our trial also demonstrated improved nutritional and anxiety status in adolescent females with PCOS who received dietary guidance from a CDSS during the 3-month intervention, in contrast to those who just received basic lifestyle instructions. It is worth noting that a significant proportion of adolescent girls in both groups displayed pronounced symptoms of anxiety prior to the intervention. PCOS is distinguished by hormonal abnormalities, specifically in insulin and androgens such as testosterone. Hormonal variations can impact neurotransmitters such as serotonin and dopamine, both essential for mood regulation in the brain [33]. Anxiety symptoms can be caused by imbalances in these neurotransmitters [33,34]. Even though we have not conducted a hormonal assessment of the participants, we could assume that the observed symptoms of anxiety can be attributed in large part to hormone dysregulation. At the same time, several studies have explored the potential role of the MD in anxiety. Most of them indicated that following this dietary pattern may have beneficial effects on mental well-being, including anxiety reduction [35]. The Mediterranean diet’s focus on nutrient-rich foods and beneficial fats likely played a role in the observed enhancements in individuals’ dietary intake, such as an increase in vitamin D, monounsaturated fatty acids (MUFA), and fiber consumption [36]. Vitamin D, with its antioxidant, neuroprotective, and neurotrophic characteristics, impacts the brain tissues which are related to the development of anxiety [37]. Accordingly, it has already been suggested that SFA intake is positively associated with anxiety disorder, while MUFA intake shows an inverse relationship with anxiety status [38,39]. In line with these studies, we can assume that the improved adherence to the MD in the MD group was associated with decreased anxiety levels.

To the best of our knowledge, this is the first trial to assess the efficacy of a CDSS in enhancing MD adherence, while at the same time exploring the effects of MD on the nutritional and anxiety status of PCOS adolescent females as indicated by anthropometry, dietary intake of nutrients, and hematology assessment including triglycerides (TG), vitamin D, and calcium status.

Nevertheless, it is important to take into account the constraints of the current trial when interpreting the results. The current trial may be subject to certain limitations, including the absence of hormonal and inflammatory assessment and cardiovascular disease (CVD) markers. To this point, there is evidence of upregulation of TG levels in adolescents with PCOS [40], while other CVD indices such as arterial blood pressure, serum total cholesterol, LDL, HDL, and fasting glucose lie within normal ranges in PCOS adolescents [41]. Moreover, there was a relatively small sample size and reliance on self-reporting instruments such as food diaries. However, these constraints were mitigated by implementing a computer-generated simple randomization. In addition, it is worth noting that all designated dietitians who supervised participants during the intervention were highly qualified. Additionally, the nutritional and psychological status instruments have undergone validation specifically in the Greek population. Finally, while a 3-month intervention period provides valuable insights into the short-term effects of dietary interventions on PCOS management, longer-term studies are needed to evaluate sustained changes in dietary habits, anthropometric measures, and psychological outcomes. Additionally, the complexity of anxiety as an outcome necessitates careful consideration of multiple factors influencing its trajectory and response to dietary interventions.

## 5. Conclusions

The main outcome of the present randomized controlled trial is the beneficial effect of a CDSS in Mediterranean diet adherence by adolescent females with PCOS. The intervention through the CDSS led to improvements in dietary habits (i.e., decreased intake of energy, total fat, and SFA, and increased intake of fibers and MUFA), body weight, and body composition, as well as anxiety. Regarding biochemical indices, TGs which play an important role in PCOS symptomatology were significantly reduced, while vitamin D and calcium levels were ameliorated. These results underscore the importance of dietary interventions tailored to the Mediterranean diet principles in the management of PCOS among adolescent females, emphasizing the potential for personalized dietary guidance and early intervention strategies through CDSS software applications.

## Figures and Tables

**Table 1:** Baseline characteristics of the 40 outpatient females with polycystic ovary syndrome of IASO hospital.

Characteristics	Individuals Enrolled (n = 40)	Control Group (n = 20)	MD Group (n = 20)	*p*-Value
**Age (yrs)** [*mean* (*SD*)]	16.2 (0.8)	16.2 (0.8)	16.3 (0.8)	0.85
**Height (m)** *mean* (*SD*)	1.66 (0.03)	1.66 (0.03)	1.66 (0.03)	0.58
**Weight (kg)** *mean* (*SD*)	61.4 (6.2)	62.8 (5.4)	60 (6.6)	0.15
**BMI (kg/m^2^)** *mean* (*SD*)	22.3 (2.1)	22.8 (1.7)	21.7 (2.3)	0.08
**Weight status based on IOTF** *n* (*%*)				
Underweight	0 (0)	0 (0)	0 (0)	-
Normal weight	40 (100)	20 (100)	20 (100)	
Overweight/obese	0 (0)	0 (0)	0 (0)	
**TEE (kcal/d)** *mean* (*SD*)	2321 (98)	2340 (89)	2303 (105)	0.233
**HADS-anxiety** *n* (*%*)				
**0–7**	17 (42.5)	7 (35)	10 (50)	0.53
**8–10**	9 (22.5)	6 (30)	3 (15)	
**>10**	14 (35)	7 (35)	7 (35)	

Values are presented as frequencies (%) or mean and standard deviation of the mean (SD). *p*-values derived from Student’s *t*-test for continuous variables or the chi-square test for the categorical variables. BMI: Body Mass Index; IOTF: International Obesity Task Force; TEE: Total Energy Expenditure; HADS: Hospital Anxiety and Depression Scale.

**Table 2:** Participants’ anthropometric characteristics and blood markers at baseline and follow-up.

Characteristics	Group	Baseline (n = 20)	Follow-Up (n = 20)	*p* ^2^
**Body weight (kg)** [mean (SD)]	Control	62.8 (5.4)	61.2 (4.9)	**<0.001**
	MD	60 (6.6)	54.7 (4.9)	**<0.001**
	***p* ^1^**	0.15	**<0.001**	
**Fat Mass (kg)** [mean (SD)]	Control	19.4 (2.3)	19.0 (2.2)	0.053
	MD	16.7 (2.5)	14.3 (2.4)	**<0.001**
	***p* ^1^**	**0.001**	**<0.001**	
**Triacylglycerols (mg/dL)** [median (IQR)]	Control	100 (69)	68 (50)	0.78
	MD	99 (59)	61 (45)	**0.002**
	***p* ^1^**	0.97	0.38	
**Ca** [mean (SD)]	Control	8.7 (0.57)	8.6 (0.57)	0.35
	MD	8.9 (0.40)	9.1 (0.49)	**0.002**
	***p* ^1^**	0.19	**0.013**	
**Vitamin D** [mean (SD)]	Control	30.8 (4.0)	32.2 (3.4)	**<0.001**
	MD	32.2 (3.7)	36.1 (5.4)	**<0.001**
	***p* ^1^**	0.28	**0.011**	

MD: Mediterranean diet; Ca: Calcium; *p*: *p*-value. Values are depicted as mean and standard deviation of the mean (SD) for variables that were normally distributed and as median (IQR) for non-normally distributed variables, *p* ^1^: between Control and MD group (independent sample *t*-test or Mann–Whitney U test), *p* ^2^: for time effect (paired-samples *t*-test or Wilcoxon signed-rank test).

**Table 3:** Participants’ dietary intake at baseline and follow-up.

Characteristics	Group	Baseline (n = 20)	Follow-Up (n = 20)	*p* ^2^
**Energy intake (kcal)** [median (IQR)]	Control	2455 (288)	2600 (575)	0.09
	MD	2400 (225)	2050 (238)	**0.015**
	***p* ^1^**	0.10	**<0.001**	
**Protein intake (g)** [mean (SD)]	Control	18.4 (3.9)	19.4 (2.1)	0.17
	MD	20.8 (3.0)	20.4 (2.0)	0.911
	***p* ^1^**	**0.039**	0.11	
**Carbohydrate intake (g)** [mean (SD)]	Control	52.6 (4.7)	52.7 (3.4)	0.50
	MD	49.6 (3.9)	50.4 (3.0)	0.49
	***p* ^1^**	**0.037**	**0.029**	
**Fiber intake (g)** [mean (SD)]	Control	20.3 (4.0)	21.2 (3.8)	**0.04**
	MD	19.6 (4.1)	25.1 (5.0)	**<0.001**
	***p* ^1^**	0.57	**0.008**	
**Total Fat intake (g)** [median (IQR)]	Control	64.5 (18.3)	68.5 (18.5)	**<0.001**
	MD	58.5 (6.5)	53.5 (6.0)	**<0.001**
	***p* ^1^**	0.16	**<0.001**	
**SFA intake (g)** [median (IQR)]	Control	17 (8.8)	17.8 (7.6)	0.30
	MD	17.4 (2.5)	14.3 (5.0)	**0.002**
	***p* ^1^**	0.74	0.06	
**MUFA intake (g)** [median (IQR)]	Control	23.2 (7.3)	24 (7.5)	0.67
	MD	28.2 (10.2)	34.2 (4.7)	**<0.001**
	***p* ^1^**	**0.02**	**<0.001**	
**Cholesterol intake (g)** [mean (SD)]	Control	187 (23)	201 (26)	**<0.001**
	MD	168 (51)	150 (44)	**<0.001**
	***p* ^1^**	0.12	**<0.001**	
**KIDMED Score** [mean (SD)]	Control	5.91 (0.8)	5.95 (0.7)	0.48
	MD	6.04 (0.9)	7.03 (0.7)	**<0.001**
	***p* ^1^**	0.63	**<0.001**	

MD: Mediterranean diet; KIDMED Score (range −4 to 12): adolescents’ adherence to the MD, SFA: Saturated Fatty Acids; MUFA: Mono-unsaturated Fatty Acids; *p*: *p*-value. Values are depicted as mean and standard deviation (SD) of the mean for variables that were normally distributed and as median (IQR) for variables non-normally distributed, *p* ^1^: between Control and MD group (independent sample *t*-test or Mann–Whitney U test), *p* ^2^: for time effect (paired-samples *t*-test or Wilcoxon signed-rank test).

## Data Availability

Data are unavailable due to privacy or ethical restriction.
